# Urinary tract infections in renal transplant recipients at a quaternary care centre in Australia

**DOI:** 10.1186/s12882-019-1666-6

**Published:** 2019-12-27

**Authors:** Simon OLENSKI, Carla SCUDERI, Alex CHOO, Aneesha Kaur BHAGAT SINGH, Mandy WAY, Lakshmanan JEYASEELAN, George JOHN

**Affiliations:** 1 Royal Brisbane and Women’s Hospital, Brisbane, QLD Australia; 2 University of Queensland, Brisbane, QLD Australia; 3 Department of Renal Medicine, Sunshine Coast University Hospital, PO Box 5340, Sunshine Coast, MC Qld 4560 Australia; 4 QIMR Berghofer Medical Research Institute, Brisbane, QLD Australia; 5 Christian Medical College, Vellore, Tamil Nadu India

**Keywords:** Antibiotics, Creatinine, Renal transplant, Risk factors, Urinary tract infection

## Abstract

**Background:**

Urinary tract infections (UTI) are the most common of infections after renal transplantation. The consequences of UTIs in this population are serious, with increased morbidity and hospitalisation rates as well as acute allograft dysfunction. UTIs may impair overall graft and patient survival. We aimed to identify the prevalence and risk factors for post-transplant UTIs and assess UTIs’ effect on renal function during a UTI episode and if they result in declining allograft function at 2 years post-transplant. Additionally, the causative organism, the class of antibacterial drug employed for each UTI episode and utilisation rates of trimethoprim/sulfamethoxazole (TMP/SMX) prophylaxis were also quantified.

**Methods:**

This was a retrospective study of 72 renal transplant patients over a 5-year period who were managed at the Royal Brisbane and Women’s Hospital. Patient charts, pathology records and dispensing histories were reviewed as part of this study and all UTIs from 2 years post transplantation were captured.

**Results:**

Of these patients, 20 (27.8%) had at least one UTI. Older age (*p* = 0.015), female gender (*p* < 0.001), hyperglycaemia (*p* = 0.037) and acute rejection episodes (*p* = 0.046) were risk factors for developing a UTI on unadjusted analysis. Female gender (OR 4.93) and age (OR 1.03) were statistically significant risk factors for a UTI on adjusted analysis. On average, there was a 14.4% (SEM 5.20) increase in serum creatinine during a UTI episode, which was statistically significant (*p* = 0.027), and a 9.1% (SEM 6.23) reduction in serum creatinine after the UTI episode trending toward statistical significance.

(*p* = 0.076). Common organisms (*Escherichia coli and Klebsiella pneumoniae*) accounted for 82% of UTI episodes with 70% of UTI cases requiring only a single course of antibiotic treatment. Furthermore, the antibiotic class used was either a penicillin (49%) or cephalosporin (36%) in the majority of UTIs. The use of TMP/SMX prophylaxis for *Pneumocystis carinii* pneumonia prophylaxis did not influence the rate of UTI, with > 90% of the cohort using this treatment.

**Conclusions:**

There was no significant change in serum creatinine and estimated glomerular filtrate rate from baseline to 2 years post-transplant between those with and without a UTI.

## Background

Urinary tract infections (UTI) are the most common of infections after renal transplantation with an estimated incidence between 10 and 98% [1, 2]. The wide variation can be attributed to various factors including inconsistent definitions and diagnostic criteria among studies as well as variable utilisation rates of prophylactic antibiotics. Consequently, the diagnosis of UTI among renal transplant recipients has been fraught by such inconsistencies up until now. Updated 2019 guidelines from the American Society of Transplantation Infectious Diseases Community of Practice have attempted to unify definitions for all UTI syndromes including: *asymptomatic bacteriuria*, *acute simple cystitis (lower UTI), acute pyelonephritis (complicated UTI),* and *recurrent UTI* [3]*.* These definitions are outlined in Table 1. The ‘classical’ symptoms of UTI, which includes urinary frequency, dysuria, urgency or suprapubic pain, are often absent in the renal transplant population due to the combination of immunosuppression and surgical denervation of the transplanted kidney and ureters [4]. As such, UTI in a renal transplant recipient may present as a new febrile illness, urosepsis or an asymptomatic rise in serum creatinine. A urine dipstick test is routine practice in the transplant outpatient setting allowing for the early detection of asymptomatic bacteriuria in transplant patients.

**Table 1:** Classification of asymptomatic bacteriuria and UTI in renal transplant recipients [3]

Classification	Description
Asymptomatic bacteriuria	No urinary or systemic symptoms of infection
Acute simple cystitis	Dysuria, urinary urgency/frequency, or suprapubic pain; but no systemic symptoms and no ureteral stent/nephrostomy tube/chronic urinary catheter
Acute pyelonephritis/complicated UTI	Fever, chills, malaise, haemodynamic instability, or leukocytosis (without other apparent etiology); flank/allograft pain; or bacteremia with same organism as in urineDysuria, urgency, frequency, suprapubic pain may or may not be present
Recurrent UTI	≥3 UTIs in prior 12 month period

Gram-negative rods are the main pathogens causing UTIs in both the non-transplant and transplant populations [5]. *Escherichia coli* is the most common uropathogen and other common enteric organisms include *Klebsiella pneumoniae, Pseudomonas aeruginosa* and *Enterococci species* [2]*.* Furthermore, on a global scale there are increasing numbers of pathogenic multi-drug resistant (MDR) gram negative bacteria. This is most prevalent and problematic in the Australasian region, as increasing travel and waves of immigration provide more opportunities for bacterial plasmids to be transferred between countries [6, 7]. MDR bacteria such as *extended-spectrum beta-lactamase (ESBL) producing Enterobacteriaceae* and *carbapenem-resistant Enterobacteriaceae* are already present in our region and often require the deployment of much older antibiotics, some of which are nephrotoxic.

Risk factors for the development of a post-transplant UTI can be divided into pre-operative (host) factors, intra-operative factors and post-operative factors. Pre-operative factors include female sex, diabetes mellitus and the presence of urological abnormalities. Intra-operative factors of note include kidney transplantation from a deceased donor, the use of ureteric stents and prolonged indwelling bladder catheterisation. Post-operative factors of note include acute allograft dysfunction and rejection as well as excessive immunosuppression as a result of rejection episodes [1, 8, 9].

The 2009 Kidney Disease Improving Global Outcomes (KDIGO) guidelines recommends TMP/SMX for prophylaxis against *Pneumocystis carinii* for at least 6 months post-transplant. More recent studies have shown a rising prevalence of resistant organisms in those with UTIs whilst on prophylaxis yet the recommendation still stands [10, 11].

The consequences of UTIs in this population are serious with increased morbidity and hospitalisation rates, as well as acute allograft dysfunction [8]. It is unclear if UTIs impair long-term allograft function or reduce allograft or patient survival, as most studies are retrospective in nature [12, 13]. A recent study of over 60,000 renal transplant recipients from 2000 to 2011 from the United States Renal Data System registry for infections in the first 12 months post-transplantation demonstrated that 32% of patients had a UTI in the first year and this was associated with a 41% increased relative risk of death [14]. Abeysekera et al’s 2018 audit of Southern Tasmanian kidney transplant recipients confirmed that UTIs are the most common infection and the most likely site of infection to require hospitalisation [15].

We aimed to identify the prevalence and risk factors for post-transplant UTIs and assess UTIs’ effect on renal function during a UTI episode. Then we assessed if UTI resulted in declining allograft function at 2 years post-transplant. We aimed to quantify which organisms were causative of UTI and collected data on classes of antibiotic employed, as well as number of different antibiotics employed per episode. Utilisation rates of TMP/SMX prophylaxis were also investigated.

## Methods

A retrospective observational study was conducted at the Royal Brisbane and Women’s Hospital (RBWH), a quaternary level hospital in Brisbane, Australia. This hospital receives transplanted patients for management in the early post-operative period. Baseline demographics were collected for all adult patients (> 18 years old) who received a renal transplant (including simultaneous kidney-pancreas transplantation) and whose post-acute transplant follow-up was provided at RBWH from July 1st 2011 to July 1st 2016. We included in this study all adult patients returning to our centre who had their post-transplant care with us for 2 years from their renal transplant date and within the above specified timeframe. We excluded those patients returning to our centre within the above timeframe but not completing their 2 years of post-transplant care with us, as well as those who received their transplant outside the specified timeframe.

For the purpose of this study, we defined UTI as the presence of bacteriuria on laboratory reports and receipt of one or more courses of antibiotics. This all-encompassing definition of UTI therefore included all cases of acute simple cystitis, transplant pyelonephritis and asymptomatic bacteriuria if treated with antibiotics. Asymptomatic bacteriuria was only treated if there was an unexplained rise in serum creatinine and deemed clinically necessary. To estimate renal function at each time-point, three consecutive sera creatinine were reviewed. ‘Pre-UTI’ was within 3 months prior to an episode, ‘UTI episode’ was within 2 weeks of a recorded bacteriuria and ‘post UTI’ was 15 days to 3 months post a UTI episode.

Patients were stratified into two groups according to the presence or absence of at least one UTI episode. All UTI episodes were included from 1 month after renal transplantation up to 2 years post-transplant. We further stratified the UTI sub-cohort for severity into those with simple cystitis versus those with complicated UTI/pyelonephritis. Patients with systemic symptoms and a clinical profile mandating admission to hospital for parenteral antibiotic therapy were defined as having a complicated UTI/pyelonephritis. This definition is consistent with new guidelines from the American Society of Transplantation [3].

The demographic details of the cohort are summarised in Table 2. Additionally, hospital and community pharmacy dispensing records of antibiotic therapy were interrogated to ascertain the number of antibiotic courses for each UTI episode, the class of antibiotic used and the number of patients who were receiving TMP/SMX prophylaxis.

**Table 2:** Demographic details

Variables	UTI (*n* = 20)	No UTI (*n* = 52)	*p* value
	n (%)	n (%)	
Age (Mean ± SD) Years	52.2 ± 12.5	43.0 ± 14.6	0.015
Gender			
Male	3 (15.0)	35 (67.3)	< 0.001
Female	17 (85.0)	17 (32.7)	
Type of transplant			
Deceased donation	12 (60.0)	43 (82.7)	0.112
Kidney-pancreas (KP)	5 (25.0)	5 (9.6)	
Live donation	3 (15.0)	4 (7.7)	
Acute rejection episodes			
Yes	2 (10.0)	17 (33.3)	0.046
No	18 (90.0)	34 (66.7)	
Pre-existing history of UTIs			
Yes	8 (40.0)	10 (19.6)	0.076
No	12 (60.0)	41 (80.4)	
Pre-existing urinary tract abnormality			
Yes	10 (52.6)	15 (28.8)	0.063
No	9 (47.4)	37 (71.2)	
Hyperglycaemia			0.037
No	7 (35.0)	34 (65.4)	
Pre and post	2 (10.0)	7 (13.5)	
Post-transplant diabetes	7 (35.0)	6 (11.5)	
KP transplant (pre only)	4 (20.0)	5 (9.6)	
TMP/SMX use post-transplant			
Yes	19 (95.0)	47 (92.2)	1.000
No	1 (5.0)	4 (7.8)	
CMV viraemia at any time point			
Yes	7 (35.0)	14 (26.9)	0.499
No	13 (65.0)	38 (73.1)	
BK viraemia at any time point			
Yes	4 (21.1)	16 (31.4)	0.395
No	15 (78.9)	35 (68.6)	

Statistical analysis was performed using STATA version 15. Categorical variables were summarised by frequencies and percentages and continuous variables by means and standard deviations or median and interquartile range for non-normally distributed variables. Associations between categorical variables were examined using Pearson’s Chi-squared test. Fisher’s exact test was used in situations where more than 20% of the cell counts were fewer than five. For non-correlated data, continuous variables were examined using an independent Student t-test or a Mann-Whitney test for non-normally distributed variables. Log-binomial logistic regression was used and the variables that were statistically significant at the bivariate analyses at *p*-values less than 0.05 were considered for adjusted analyses.

This study was approved by the Royal Brisbane and Women’s Hospital Human Research Ethics Committee (approval: HREC/17/QRBW/371).

## Results

Over a 5 year period, 72 patients were reviewed post-transplant. Our centre receives transplant patients from other transplanting centres and over this time-frame there were 55 deceased donor transplants, 7 living related kidney transplants and 10 simultaneous kidney-pancreas transplants. All 72 patients include in this study had received their first renal transplant.

Induction therapy consisted of either intravenous basiliximab or thymoglobulin and methylprednisolone, together with tacrolimus and mycophenolate. 95.7% (66/69) received intravenous basiliximab as their induction agent, with only 3 patients receiving thymoglobulin induction. There was no association with UTI for those that received thymoglobulin induction (*p* = 0.20). Patients were then maintained on a combination of tacrolimus, mycophenolate and prednisolone throughout the post-transplant period. Tacrolimus was dosed to target a trough of 5 ng/ml for patients with average immunological risk and 6–7 ng/ml for those with high immunological risk. Prednisolone was tapered to 6–7 mg / day wherever possible. Mycophenolate mofetil/sodium remained at 1 g twice daily/720 mg twice daily unless there was leucopenia, intolerable gastrointestinal side-effects, a high risk of infections or a new malignancy, in which case it was reduced.

The mean age at transplantation was 45.5 years (median: 47, range: 17–71). Congenital/genetic (21/71 = 29.6%) and metabolic/vascular (21/71 = 29.6%) were equally the most common cause of end-stage kidney disease leading to transplantation, with glomerulopathy (20/71 = 28.2%) also common. All patients had an indwelling catheter to measure urine output and this was routinely removed 3–5 days after transplantation. All patients had a ureteric stent inserted and this was routinely removed 4–6 weeks after transplantation unless patients developed early UTI, in which case it was removed earlier.

20 patients experienced at least one UTI over the study period and the incidence rate for a UTI was 27.8% across the whole cohort. A total of 77 UTI episodes were analysed in the UTI sub-group and the mean number of UTIs per person in this sub-group was 3.85. (Range 1–16). Specific details about UTI episodes are summarised in Table 3. 55% of the patients (11/20) in the UTI sub-group had at had at least one episode of complicated UTI/pyelonephritis. The remaining 45% (9/20) in this sub-group had at least one simple UTI episode. 33.8% of the UTI episodes were classified as a complicated UTI/pyelonephritis (26/77). Furthermore, only 8% (6/77) of blood cultures obtained at time of a UTI episode were positive and identical to the urinary isolate.

**Table 3:** UTI episodes (*n* = 77)

Most common organisms	
*E.coli*	41 (53%)
*Klebsiella species*	22 (29%)
*Pseudomonas aeruginosa*	4 (5%)
*Enterococcus faecalis*	3 (4%)
*ESCAPPM organisms*	3 (4%)
Culture negative	2 (3%)
Other	2 (3%)
Number of antibiotics received per episode	
One	54 (70%)
Two	18 (23%)
Three	5 (6%)
Class of antibiotics used	
Penicillins	38 (49%)
Cephalosporins	27 (36%)
Fluoroquinolones	12 (16%)
Carbapenems	3 (4%)
Other	14 (18%)

We isolated an *ESBL*-producing organism in 3.9% of urine cultures (3/77). The *ESBL* organism was an *E.coli* on all three occasions and all three isolates were sensitive to penicillin, gentamicin and carabepenem initially, however one of the isolates has since become carbapenem-resistant.

TMP/SMX prophylaxis at a dose of 400/80 mg/day was almost universal in our cohort (66/71 = 93%). Those not treated with TMP/SMX were due to side effects or allergy. Additional prophylactic antibiotics were received by 35% of the UTI sub-group (7/20) as an adjunct to TMP/SMX prophylaxis. 85.7% of these patients (6/7) used nitrofurantoin, of which 1 patient used methenamine hippurate in conjunction with nitrofurantoin, and 1 used cyclical cephalexin and fosfomycin. The mean number of UTIs in this sub-group of patients using additional prophylactic antibiotics was 6.86.

On univariate analysis, older age (*p* = 0.015), female gender (*p* < 0.001), hyperglycaemia (*p* = 0.037) and acute rejection episodes (*p* = 0.046) were all risk factors for developing UTI. Of note, the type of transplant, greater number of HLA mismatches and a pre-existing history of UTIs were not identified as risk factors. Furthermore, on adjusted analysis, female gender (OR 4.93, *p* = 0.007) and age (OR 1.03, *p* = 0.042) were statistically significant risk factors for developing a UTI. All other variables were not statistically significant.

**Fig. 1:**
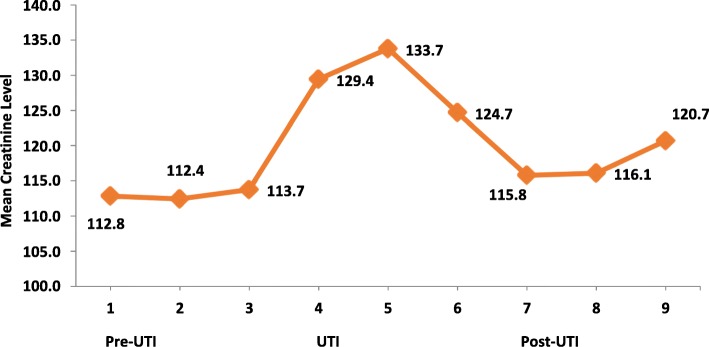
Mean serum creatinine across all 77 UTI episodes. Serum creatinine: measured in units micromol/L

With each UTI episode, there was a mean increase in serum creatinine of 21 micromol/L (14.4%, SEM 5.20) which was statistically significant (*p* = 0.027). After a UTI episode there was a mean 16 micromol/L (9.1%, SEM 6.23) reduction in serum creatinine, trending toward statistical significance (*p* = 0.076). This is summarised in Fig. 1. Overall, there was no significant change in serum creatinine and estimated glomerular rate (eGFR) from baseline out to 2 years post-transplant between those with and without a UTI. There was also no statistically significant difference in change in serum creatinine from baseline out to 2 years post-transplant between those with a complicated UTI/pyelonephritis versus those with a simple UTI (*p* = 0.331). There was no statistically significant difference in change in serum creatinine from baseline out to 2 years post-transplant between those with a complicated UTI/pyelonephritis versus the rest of the whole cohort (*p* = 0.814).

(*p* = 0.814).

## Discussion

Our study highlights the incidence of UTIs in a renal transplant population with 27.8% of this cohort experiencing a UTI. Similar rates have been demonstrated in other studies and support the notion that UTI remains the most common infection after renal transplantation. This study is informative about UTIs in a carefully followed low immunosuppression transplant population and our observations are valid only for a similar population.

The study identified risk factors for developing a UTI, many of which have been demonstrated in other studies. Female gender and age were the only risk factors that reached statistical significance on the adjusted analysis and surprisingly, a pre-existing history of UTI or urogenital abnormality were not found to be a risk factors on the unadjusted analysis. Many studies have revealed an association between stent usage and higher rates of UTI [16]. Interestingly**,** a 2016 study demonstrated that early ureteric stent removal at 1 week was associated with a statistically significant lower rate of UTIs compared with routine stent removal at 4 weeks [17].

We had very low rates of UTI or bacteremia caused by *ESBL*-producing organisms and none of our patients died as a result of UTI. Nevertheless, there is a known association between mortality and bacteremia secondary to MDR gram negative infections in transplant recipients and MDR infections are expected to become more problematic in the future [18].

Furthermore, trimethoprim usage for *Pneumocystis* prophylaxis did not confer protection against UTIs in our patients, despite 93% of patients using this treatment at doses considered prophylactic for UTIs. This raises interesting questions regarding the therapeutic effectiveness of trimethoprim for UTI prophylaxis in the kidney transplant population. Trimethoprim is also known to cause a reversible increase in serum creatinine by inhibiting the tubular secretion of creatinine [8]. As expected there was no significant difference in baseline serum creatinine levels between the two subgroups as the vast majority of patients were on TMP-SMX prophylaxis.

In this study, UTIs acutely affected renal function during an episode, with renal function approaching baseline from 2 weeks post UTI. Importantly, UTIs did not impair overall renal function at 2 years post-transplant. This is, in part, due to a broad definition of UTI encompassing patients who were clinically considered to require treatment and not just those who were symptomatic. Due to early detection and aggressive treatment of clinically significant asymptomatic bacteriuria, we yielded a milder profile of UTIs in our cohort, with only 8% of all UTI episodes having a co-existing bacteremia and no statistically significant difference in renal function at 2 years post-transplant for those experiencing a complicated UTI. Similar cohort studies have demonstrated higher rates of complicated UTI/pyelonephritis ranging from 13 to 24% [13, 19, 20].. Furthermore, in other studies severe UTIs and urosepsis may indeed have an adverse impact on long-term renal function [8]. The time-point of 2 years post-transplant was chosen as a reference point for a number of reasons. Firstly, there is a well documented increased risk of UTIs in the initial 12 months post-transplant, more specifically, in the initial three to 6 months, due to the surgical procedure itself and high levels of immunosuppression employed in this period [21, 22]. Secondly, from a pragmatic perspective, 2 years represented a reliable and accessible dataset in terms of dispensing and medical record accuracy.

Many of the reported risk factors for UTIs in this population, such as gender and age, cannot be mitigated. Moreover, vigilance and monitoring for hyperglycaemia in the transplant population is important for cardiovascular benefits aside from hyperglycaemia being a predictor of increased UTI risk. What is not clear from this study is the manner in which monitoring and surveillance of UTIs should occur, given the relative ineffectiveness at reducing UTIs demonstrated by trimethoprim prophylaxis and the non-significant difference in renal function at 2 years post-transplant across the whole cohort and between the simple UTI and complicated UTI sub-groups. What is also not clear from this study is the effectiveness of prophylactic antibiotics in those patients with frequent or recurrent UTIs, as this sub-group of UTI patients were observed to have a greater number of UTI episodes (6.86 vs UTI group mean of 3.85).

Our study adds to the growing body of work around UTI management in the kidney transplant population, by highlighting that UTIs are very common. Risk factors were in line with other published studies with female gender, older age and hyperglycaemia conferring risk. In our population, UTIs did not affect renal function at 2 years post-transplant, and despite 93% of our patients taking TMX-SMX prophylaxis, the trimethoprim component seemed not to confer protection against UTIs. Moreover, ESBL organisms causing UTIs were relatively uncommon at 3.8%. We acknowledge the limitations inherent in this study design, specifically its retrospective nature, small sample size and relatively limited follow-up. It is difficult to generalise on patients’ long-term allograft function using 2 year post-transplant data, but the results are nevertheless indicative of important trends. Finally, we acknowledge that we employed an all-encompassing UTI definition which included all cases of clinically significant asymptomatic bacteriuria, cystitis and transplant pyelonephritis; however, this is representative of current treatment practices for transplant patients. We acknowledge that the evidence does not support treating asymptomatic bacteriuria in its own right, however many clinicians would treat if it is accompanied by an unexplained rise in serum creatinine [23].

In conclusion, our results are valid for a similar closely monitored low immunosuppression transplant cohort and our findings offer an interesting perspective on the risk factor profile and treatment of UTIs as well as the concept of antibiotic prophylaxis for UTIs.

## Data Availability

The datasets used and analysed during the current study are available from the corresponding author on reasonable request.
